# Psychiatric Emergencies of Asylum Seekers; Descriptive Analysis and Comparison with Immigrants of Warranted Residence

**DOI:** 10.3390/ijerph15071300

**Published:** 2018-06-21

**Authors:** Georgios Schoretsanitis, Sarah Eisenhardt, Meret E. Ricklin, David S. Srivastava, Sebastian Walther, Aristomenis Exadaktylos

**Affiliations:** 1 University Hospital of Psychiatry, 3008 Bern, Switzerland; saraheisenhardt11@googlemail.com (S.E.); sebastian.walther@puk.unibe.ch (S.W.); 2 Department of Psychiatry, Psychotherapy and Psychosomatics, and JARA–Translational Brain Medicine, RWTH Aachen University, 52074 Aachen, Germany; 3 Department of Emergency Medicine, Inselspital, University Hospital Bern, Freiburgstrasse, 3010 Bern, Switzerland; meret.ricklin@gmail.com (M.E.R.); DavidShiva.Srivastava@insel.ch (D.S.S.); Aristomenis.Exadaktylos@insel.ch (A.E.)

**Keywords:** asylum seekers, psychiatric emergency services, involuntary treatment, psychiatric hospitalization

## Abstract

*Background:* The aim of our study was to assess utilization patterns of psychiatric services by asylum seekers. *Methods:* We included 119 adults who presented themselves at the University Emergency Department between 1 March 2012 and 1 January 2017 for psychiatric consultation. Descriptive data were compared with a control group of non-Swiss individuals with warranted residence permits using Mann-Whitney-*U* and chi square (χ^2^) tests. *Results:* Patients were mainly single, male, residing in reception centers, and presented themselves most frequently due to suicidal ideation. Almost 60% of the patients were assigned to inpatient treatments, with 28 involuntary cases. Compared to the control group, asylum seekers were younger and more often men (*p* < 0.001 for both). Further, they less often had family in Switzerland (χ^2^ = 9.91, *p* = 0.007). The proportion of patients coming in as walk-ins was significantly higher in the control group than in asylum seekers (χ^2^ = 37.0, *p* < 0.001). Asylum seekers were more frequently referred due to suicidal ideation and aggressive behavior than participants in the control group (χ^2^ = 80.07, *p* < 0.001). Diagnoses for asylum seekers infrequently included mood, as they often reported stress-related disorders (χ^2^ = 19.6, *p* = 0.021) and they were infrequently released home (χ^2^ = 9.19, *p* = 0.027). *Conclusion:* Asylum seekers more frequently demonstrated severe symptoms such as suicidal ideation and aggressive behavior and they were mainly treated as inpatients, potentially due to minimal social resources.

## 1. Introduction

The movement of people currently observed worldwide is comparable in size to the migration during/after the Second World War [1]. In Switzerland, the annual number of asylum seekers from 2008–2013 has remained stable between 40,000 and 43,000 per year, but has substantially increased during the following two years to over 65,000 new applications per year [2].

The vulnerability of asylum seekers and generally displaced persons in terms of mental health issues has been consistently described [3,4]. Despite the methodological inconsistency, there are several studies that suggest an enhanced prevalence of post-traumatic stress disorder and depression among refugees [5]. The factors that account for the deleterious effects of the refugee experience include pre-, peri- and post-migration adversities [6,7,8]. 

Epidemiological data of psychiatric emergency services’ usage by refugees are now increasingly available and provide a valuable indication of the problems refugees experience [9,10,11,12]. This evidence may be instrumental in evolving strategies for improvement of psychiatric services. At the same time, major challenges such as cultural and language barriers need to be addressed [7,13]. Utilization patterns among immigrants in general have already been assessed in the Swiss context [14,15,16]. Nevertheless, data for asylum seekers are particularly scarce. But information is strongly needed, since refugees and asylum seekers, often considered as a homogenous sample, present prominent differences regarding the psychopathology patterns and prevalence of mental disorders [10,17,18,19]. Differences also refer to the living arrangements, since asylum seekers in Switzerland mainly, but not exclusively, live in asylum centers. Detention in reception centers has been also connected with a considerable mental health burden [20]. Nevertheless, the majority of claimants face particular problems in accessing mental health services, and frequently, the only available option is a psychiatric consultation in the emergency department. Moreover, it is absolutely worthwhile to study psychiatric emergencies among asylum seekers following the rise in annual numbers of asylum seekers during the past few years (2015–2016). This increase enables a larger-scale epidemiological research, which can focus on particular dimensions of the mental healthcare for asylum seekers, such as the order of compulsory treatment. Such epidemiological data exist mainly for heterogeneous samples of patients, poorly defined as of non-Western or black origin or/and lacking further information of residency status [21,22,23,24]. 

The main aim of this study was to provide first descriptive data on usage of psychiatric emergency services by asylum seekers. We also compared categorical outcomes between asylum seekers and immigrants with residence permits to identify factors that may distinguish these two groups of non-Swiss people.

## 2. Materials and Methods

**Figure 1:**
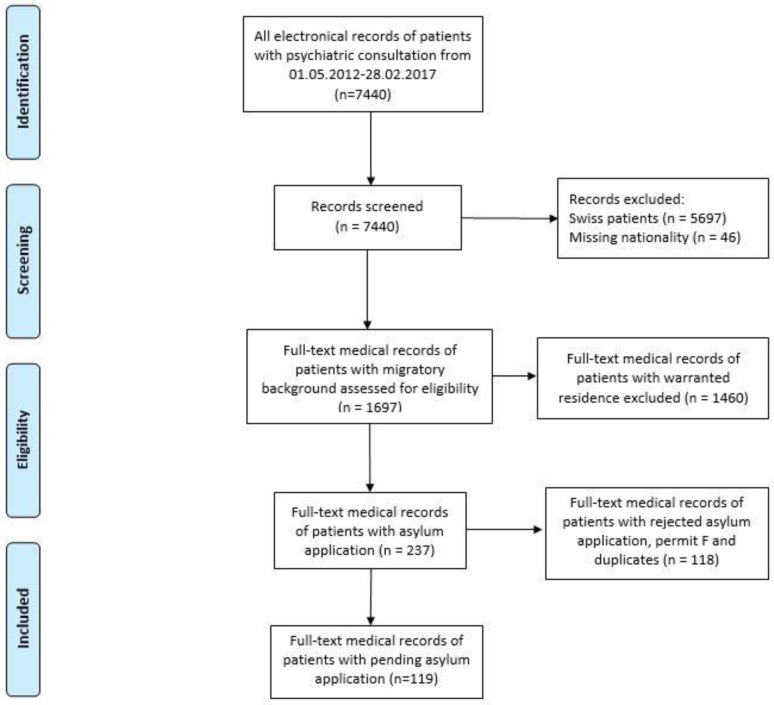
Medical records included in the analysis.

This descriptive study included retrospective data from adult asylum seekers (age ≥ 18 years). These individuals were admitted to the University Emergency Department (UNZ) for a psychiatric consultation between the 1 May 2012 and 28 February 2017. The UNZ consists of an organization, which provides 24 h/day psychiatric emergency services and it is responsible for the emergency mental healthcare for the Canton Bern (roughly 1 million citizens) including asylum centers in this catchment area. Consultations were conducted by resident doctors (medical doctors during the psychiatry training program) under supervision of senior doctors. Electronic medical records of patients with migratory background (i.e., non-Swiss nationality) were initially identified. These medical records have fastidiously been scrutinized for asylum seekers leading to 1697 records of patients with migratory background, i.e., non-Swiss nationality. Across asylum seekers there were individuals with pending cases (permit N), persons with rejected asylum application and provisionally admitted refugees (permit F). The last category consisted of foreign nationals, ordered to return to their native countries, but, in those cases, such returners were not admissible, reasonable or possible [25]. These last two subgroups experience different stressors, and were, therefore, also excluded, yielding a final sample of 119 patients (See Figure 1). During the screening process, we identified 1460 medical records of patients with migratory background and warranted residence permits. After removing duplicates and rudimentary records, we identified 1104 patients, who were included in the analysis as the control group.

We were able to extract the following demographic and clinical data from the included medical records: gender, age, nationality, marital status, children, type of residence (reception center or not), the pathway to care (health professionals involved in the referral), the reason for presentation/referral, diagnosis at the time of consultation (according to ICD-10), referral outcome, presence of interpreter during the consultation and contact with psychiatric services prior to the recorded (current) consultation. While classifying patients according to their pathway to care, the following categories were formed: walk-in, general practitioner or other medical doctor (MD), ambulance, police, or reception centers. Note that during the asylum process, the Swiss government provides healthcare coverage on a mandatory basis; everyone is entitled access to a general practitioner. The reasons for presentation/referral were grouped as follows: suicidal thoughts, suicide attempt, auto-aggressive behavior, aggression, mood (depression) symptoms, sleep disorders, acute stress, somatic complaints, psychotic symptoms, psychosocial problems and medication acquisition. If a patient has been registered for more than one reason, the major concern at the time of presentation/referral was used as the main criterion for the outcome. In particular, when suicidal and depressive symptoms co-existed, patients were classified as referred due to suicidal ideation since they were more likely to be referred mainly due to the suicidal than depressive symptoms. Therefore, all patients classified as having depressive symptoms were unlikely to have reported suicidal ideation. Following up on the referral process the possible outcomes were admittance to a psychiatric clinic (voluntary or compulsory) or discharge home. Individuals with rudimentary case files or incomplete medical records were excluded from the analysis. Moreover, for patients who visited the UNZ more than once, only the most recent registration was considered. Based on a rough geographical classification, the origin countries have been formed into six groups; Middle East (Iran, Iraq, Jordan, Lebanon, Syria, Turkey, United Arab Emirates), Eastern Europe (Albania, Belarus, Bosnia-Herzegovina, Chechnya, Macedonia, Kosovo, Russia), sub-Saharan Africa (Angola, Benin, Congo, Eritrea, Ethiopia, Guinea, Nigeria, Somalia, Sudan, Togo), northwestern Africa (Algeria, Libya, Morocco, Tunisia), Central-South Asia (Afghanistan, Bangladesh, China, Sri Lanka). 

The study was performed retrospectively with health-related patient data that were exported anonymously for analysis. One of the authors (MER) had access to identification patient information and none of the authors was potentially a treating physician of the patients involved. Retrospective analysis of data for this study was in accordance with the local regulatory authority with the Declaration of Helsinki. No informed consent was necessary for this type of study.

## 3. Statistical Analysis

The data were summarized using descriptive statistics (means and standard deviation). We assessed gender, age, type of residence, referral conditions (reason and referring person), presence of an interpreter and prior contact to psychiatric services. Diagnoses (ICD-10) were classified into diagnostic categories (F10-19, 20-29, 30-39, 40-49, F60-69, F90-99). Demographic characteristics and parameters related to the registered consultation were compared between the group of asylum seekers and a control group of non-Swiss patients with warranted permits: our primary hypothesis was that involuntary treatment orders would be increased in the group of asylum seekers due to issues of integration; we studied referral outcomes as a categorical variable, but we also transformed referral outcomes to a dichotomous variable with ‘0’ for cases of non-compulsory treatment consisting of discharge home and voluntary admissions in the clinic. We used the variable ‘1’ for compulsory treatment consisting of involuntary admissions. The comparison between study groups was based on non-parametrical tests Mann-Whitney-*U* (M-W-*U*) test and the chi square test (χ^2^) with a significance level of *p* < 0.05. For the computation of possible correlations, cases without data were excluded. All statistical analyses were carried out using IBM SPSS Statistics, version 22.0 (IBM GmbH, Ehningen, Germany).

## 4. Results

The medical records were scanned for asylum seekers leading to a sample of 119 persons (30 women, 89 men). The mean age was 29.88 ± 9.13 (range 18–57) years (Table 1). In terms of origin, individuals came from a wide range of countries; the most common origin countries were Eritrea (*n* = 17), Afghanistan (*n* = 14) and Morocco (*n* = 12) (Table 2). The majority of the patients were single (*n* = 67, 56.3%) and 72 of the patients did not have children (60.5%). Almost 6 out of 10 patients (*n* = 66, 55.5%) had no relatives in Switzerland. Asylum seekers lived mainly in reception centers (*n* = 85, 71.4%).

Predominantly, patients came as walk-ins patients (*n* = 52, 43.7%), whereas 35 patients (29.4%) were referred to the UNZ by the police (Table 3). The most frequent reason of referral was suicidal ideation, reported by 30 patients (25.2%), followed by aggressive behavior, which was the referral reason for 25 patients (21.0%). Seventy patients reported prior contact to psychiatric services (58.8%). The consultation was aided by interpreting service in 29 cases (24.4%) or by the presence of a person who was a speaker of the patient’s native language (family member or colleague of the patient) in 9 cases (7.6%), which was not possible in two thirds of the patients (*n* = 80, 67.2%).

The diagnostic picture is dominated by adjustment disorders (F43.2), which comprised one fourth of the diagnoses (*n* = 30, 25.21%). Twenty-two patients (18.5%) received a diagnosis of schizophrenia spectrum disorders, whereas patients were diagnosed with affective and substance use disorders in 14.3% (*n* = 17) and 11.8% (*n* = 14) of the cases, respectively. Regarding the referral outcomes, 59.7% (*n* = 71) of patients were treated as inpatients: 42 voluntary treatment cases and 29 compulsory treatment cases (Table 3). 

The group of asylum seekers was compared with a control group of non-Swiss citizens with warranted residence permits in terms of demographic/clinical characteristics and consultation aspects (Table 1). The two groups showed significant differences for age and sex distribution; asylum seekers were younger (*p* < 0.001 for M-W-*U*), more often men (χ^2^ = 17.64, df = 1, *p* < 0.001) and less likely to have been married than participants in the control group (χ^2^ = 22.57, df = 5, *p* < 0.001). No differences between groups were reported in the proportion of patients having children; nevertheless, the proportion of asylum seekers with children abroad was higher than in the control group and asylum seekers less frequently reported children living in Switzerland than patients in the control group (χ^2^ = 22.3, df = 3, *p* < 0.001). Patients in the control group more frequently had relatives residing in Switzerland compared to asylum seekers (χ^2^ = 9.91, df = 2, *p* = 0.007).

Moreover, the two groups differed regarding referral conditions; the proportion of patients presenting as walk-ins was significantly higher for patients of the control group than asylum seekers (χ^2^ = 37.0, df = 4, *p* < 0.001). The rest of the referral conditions did not differ between groups (*p* > 0.05 for χ^2^ except from referral by the reception center, where no counts were available in the control group and, therefore, no valid analysis was possible. When exploring potential differences in referral reasons between groups, we detected higher rates of suicidal ideation and aggressive behavior but lower rates of depressive and psychotic symptoms in asylum seekers (χ^2^ = 80.07, df = 15, *p* < 0.001 for all comparisons). No differences were reported for somatic complaints (*p* > 0.05), whereas analyses were not conducted for the rest of reasons due to small number of counts per reason and group. The ICD diagnoses set by consulting psychiatrists differed for mood disorders (F30-39) and stress-related disorders (F40-49), with the mood disorders occurring proportionally less often and the stress-related disorders occurred proportionally more often in asylum seekers compared to control group (χ^2^ = 19.6, df = 9, *p* = 0.021). Regarding referral outcome, the proportion of asylum seekers released home was lower than in the control group (χ^2^ = 9.19, df = 3, *p* = 0.027). For assignment to compulsory treatment (using referral outcome as a binary variable), study groups did not demonstrate differences (χ^2^ = 0.85, df = 1, *p* > 0.05).

## 5. Discussion

Our study provides a descriptive and comparative analysis of utilization of psychiatric services for asylum seekers with pending application. To our knowledge, our retrospective assessment adds to previous research because access to mental healthcare for immigrants remains scarce. Nevertheless, this particular subgroup has distinct mental health needs as these patients are exposed to additional mental health risks [26]. Our study, contrasting previous studies, uses the migration status rather than the country of birth or race as a determinant for mental health problems. To our knowledge, specific parameters, such as the order of a compulsory treatment for these individuals, were examined, whereas the main point widely evaded the focus of research. In this context, we chose to include all psychiatric diagnoses, while most of earlier studies focused on refugees with schizophrenic disorders, and particularly first-episode patients. Moreover, the criteria for compulsory admissions largely vary between and even within countries since they strongly depend on the current mental health legislation [27].

The profile of asylum seekers in our sample indicated younger and more often male individuals compared to the control group. They were mainly residing in reception centers and they had no family in Switzerland. Less than the half of patients presented themselves as walk-ins to the UNZ and the most common referral reason was suicidal ideation. Consultants most frequently set a stress-related diagnosis and arranged an inpatient treatment in 40% of the cases. Asylum seekers reported less family support with fewer relatives in Switzerland than controls.

Moreover, the two groups differed regarding referral conditions; the proportion of patients coming in as walk-ins was significantly higher in the control group than asylum seekers. Asylum seekers were consulted more often due to suicidal ideation or aggressive behavior, but less often for psychotic or depressive symptoms compared to controls. Mood disorders were diagnosed in a higher proportion of patients in the control group than in asylum seekers, whereas the rates of stress-related disorders were higher for asylum seekers. Finally, the proportion of individuals released home was smaller in the asylum seekers than in the control group and no differences were reported for assignment to compulsory treatment.

When interpreting these differences, the age difference between study groups seems to be a plausible finding. Regarding the difference pertaining to gender, previous evidence indicated female underrepresentation [11,16,28,29]. In our sample, this finding may be due to the two-fold higher percentages of men in asylum seekers during the years 2011–2015 [2]. Moreover, the high percentage of aggressive behavior as referral reason (almost one out of fourth) for asylum seekers may be accounted for by gender, as aggression is more common in men than in women [30]. The proportion of individuals consulted for suicidal ideation was higher for asylum seekers than for controls; a Danish psychiatric emergency service study also reported suicidal ideas as the most common referral reason for asylum seekers [11]. Likewise, hospital data reported high incidence of suicidal behavior in asylum seekers residing in reception centers in the Netherlands [31]. On the other hand, the low rates of asylum seekers referred for depressive symptoms may be due to the overrepresentation of stress-related disorders in our sample as well as in previous samples [7,17,32], as stress-related disorders may imitate depression in terms of psychopathology [33]. Moreover, an essential amount of asylum seekers with depressive symptoms was classified in the subgroup referred for suicidal ideation. Thus, it would be more precise to say that rates of depressive symptoms without co-existing suicidal ideation were higher in the control group. Further, asylum seekers were less often visiting the UNZ as walk-ins; a previous Irish study reported that asylum seekers tended to visit general practitioners, who may act as primary mental healthcare providers, more frequently than did controls [17]. Alternatively, we speculated that asylum seekers are less familiar with the structure of the health care system in Switzerland, so that they might hesitate to directly visit the UNZ. For both options, the stigmatizing impact of psychiatry may account for the barriers of these individuals to access to mental healthcare [34]. Nevertheless, no differences were reported regarding alternative pathways to mental health care between groups. The finding of high prevalence of stress-related disorders in terms of set diagnoses is no surprise but is in alignment with previous evidence [11,17]. Further, it is also rather expected that a higher proportion of asylum seekers were assigned to inpatient treatment, which may be understood in light of the fact that they showed more severe symptoms such as suicidal ideation and aggressive behavior than the control group. Moreover, controls may have more social resources due to relatives also residing in the country. Thus, the limited social support may account for the higher rates of asylum seekers treated as inpatients. The assignment to inpatient treatment may also relate to the access problems to specific treatments for asylum seekers, which was reported previously in cohort studies, also in the Swiss context [14,35].

Asylum status failed to correlate with assignment to compulsory treatment in our sample. This major counter-intuitive finding introduces a riddle that may demand quantitative data to unravel mediating mechanisms. In a sample of unaccompanied refugee minors with insecure asylum status, researchers reported high rates of involuntary treatment for these individuals referred due to self-harm and suicidal behavior [32]. Nevertheless, in our sample, differences for involuntary treatment between study groups did not reach statistical significance despite the high prevalence of suicidal ideation for asylum seekers.

Anxiety levels for asylum seekers may be strongly related to language issues [29]. The usage of interpreting services in our sample was very low compared to a Danish study [11], which may introduce an important challenge for the improvement of the mental healthcare system. The ethnic distribution of the asylum seekers presented in the emergency department reflects the mosaic of ethnicities of people applying for asylum in the Canton of Bern during the past year (2016). The countries with the most applications included Afghanistan, Eritrea, Syria and Iraq [2].

Due to the retrospective analysis of this database, results must be interpreted with some caution. No standardized general and systemic medical history was taken. Parameters such as socio-economic status, length of stay in Switzerland before presentation, symptom severity and illness onset and duration, were barely provided and therefore could not be included in the analyses. Moreover, asylum seekers in Switzerland invariably are not allowed to work. As a result, we were not able to control for the effects of this variable, although the role of unemployment for compulsory admissions as well as for the psychopathology already has been demonstrated [29,36]. In addition, no data regarding the length of asylum procedure were provided; nevertheless, this parameter has been associated with an increased risk of mental disorders [37,38,39]. Lastly, the control group was not matched for demographic or clinical characteristics with the group of asylum seekers.

## 6. Conclusions

Concluding, our data imply that persons presented for psychiatric consultation had severe symptoms and were more likely than persons with permanent permit to be treated as inpatients. The treatment of this distinct patient subgroup introduces a public mental health challenge that needs to be addressed urgently. We strongly hope that this study may inspire the conduction of prospective studies providing a better overview of the mental health of asylum seekers and enabling the minimization of the application of compulsory admissions.

## Figures and Tables

**Table 1:** Sociodemographic characteristics of asylum seekers (*n* = 119) and control group (*n* = 1104).

	Asylum Seekers	Control Group
Females (%)	30 (25.2) *	500 (45.3)
Age (SD)	29.88 (9.13) **	36.98 (12.81)
Marital status (%)		
Never been married	67 (56.3) ***	458 (41.5)
Widowed & Divorced	9 (7.5)	210 (19.0)
Married (Spouse in CH)	13 (10.9)	227 (20.6)
Married (Spouse abroad)	11 (9.2)	101 (9.1)
Unknown	19 (16.0)	108 (9.8)
Children (%)		
No	72 (60.5)	609 (55.2)
Yes (Children in CH)	18 (15.1) ****	348 (31.5)
Yes (Children abroad)	10 (8.4) ****	33 (3.0)
Unknown	19 (16.0)	114 (10.3)
At least one relative in CH, not partner (%)		
No	66 (55.5)	521 (47.2)
Yes	34 (28.6) *****	470 (42.6)
Unknown	19 (16.0)	113 (10.2)

* χ^2^ = 17.64, df = 1, *p* < 0.001, ** *p* < 0.001 for M-W-*U*, *** χ^2^ = 22.57, df = 5, *p* < 0.001, **** χ^2^ = 22.3, df = 3, *p* < 0.001, ***** χ^2^ = 9.91, df = 2, *p* = 0.007.

**Table 2:** Regions of origin for asylum seekers (*n* = 119).

Regions of Origin (%)	
Middle East	27 (22.9)
Eastern Europe	11 (9.3)
Sub-Saharan Africa	33 (28.0)
Northwestern Africa	26 (22.0)
Central-South Asia	21 (17.8)

**Table 3:** Clinical characteristics of asylum seekers (*n* = 119) and control group (*n* = 1104).

Variable	Asylum Seekers	Control Group
Pathway to UNZ (%)		
Walk-in	52 (43.7) *	653 (59.1)
General practitioner (or other MDs)	16 (13.4)	101 (9.1)
Ambulance	13 (10.9)	105 (9.5)
Police	35 (29.4)	245 (22.2)
Reception center	3 (2.5)	NA
Referral reason		
Suicidal ideation	30 (25.2) **	122 (11.1)
Suicide attempt	3 (2.5)	43 (3.9)
Auto-aggressive behavior	7 (5.9)	15 (1.4)
Aggressive behavior	25 (21.0) **	103 (9.3)
Psychotic symptoms	11 (9.2) **	186 (16.8)
Depressive symptoms	10 (8.4) **	172 (15.6)
Sleep disorders	8 (6.7)	60 (5.4)
Acute stress	9 (7.6)	91 (8.2)
Somatic complaints	10 (8.4)	73 (6.6)
Psychosocial problems	2 (1.7)	186 (16.8)
Medication acquisition	2 (1.7)	7 (0.6)
Manic symptoms	0 (0)	9 (0.8)
Addiction	0 (0)	23 (2.1)
Other	2 (1.7)	14 (1.3)
ICD diagnoses		
Disorders due to substance use (F10-19)	14 (11.8)	153 (13.9)
Disorders of schizophrenia spectrum (F20-29)	22 (18.5)	243 (22.0)
Affective disorders (F30-F39)	17 (14.3) ***	256 (23.2)
Stress-related disorders (F40-F49)	58 (49.6) ***	398 (36.1)
Personality disorders (F60-69)	6 (5.0)	26 (2.4)
Others	1 (0.8)	23 (2.1)
Referral outcome (%)		
Discharged home	32 (26.9) ****	453 (41.0)
Discharged home with outpatient treatment	16 (13.4)	110 (10.0)
Voluntary admission	42 (35.3)	312 (28.3)
Compulsory admission	29 (24.4)	229 (20.7)

NA: not applicable, * χ^2^ = 37.0, df = 4, *p* < 0.001, ** χ^2^ = 80.07, df = 15, *p* < 0.001, *** χ^2^ = 19.6, df = 9, *p* = 0.021, **** χ^2^ = 9.19, df = 3, *p* = 0.027.
